# Harmonized pretreatment quantitative volume-based FDG-PET/CT parameters for prognosis of stage I–III breast cancer: Multicenter study

**DOI:** 10.18632/oncotarget.27851

**Published:** 2021-01-19

**Authors:** Kazuhiro Kitajima, Yasuo Miyoshi, Tetsuro Sekine, Hiroyuki Takei, Kimiteru Ito, Akihiko Suto, Hayato Kaida, Kazunari Ishii, Hiromitsu Daisaki, Koichiro Yamakado

**Affiliations:** ^1^Department of Radiology, Hyogo College of Medicine, Hyogo, Japan; ^2^Department of Breast and Endocrine Surgery, Hyogo College of Medicine, Hyogo, Japan; ^3^Department of Radiology, Nippon Medical School Hospital, Tokyo, Japan; ^4^Department of Breast Surgery and Oncology, Nippon Medical School Hospital, Tokyo, Japan; ^5^Department of Diagnostic Radiology, National Cancer Center Hospital, Tokyo, Japan; ^6^Department of Breast Surgery, National Cancer Center Hospital, Tokyo, Japan; ^7^Department of Radiology, Kinki University Faculty of Medicine, Osaka, Japan; ^8^Department of Radiological Technology, School of Radiological Technology, Gunma Prefectural College of Health Sciences, Maebashi, Japan

**Keywords:** breast cancer, survival, fluorodeoxyglucose (FDG), positron emission tomography-computed tomography (PET-CT), harmonization

## Abstract

Objectives: This study investigated harmonized pretreatment volume-based quantitative FDG-PET/CT parameters in breast cancer patients for prognostic value.

Results: During a median overall follow-up period of 5.3 years, 91 patients had recurrence and 40 died. Multivariate analysis of ER-positive/HER2-negative patients showed high maximum standardized uptake value (SUVmax) (*p* = 0.018), high total lesion glycolysis (TLG) (*p* = 0.010), and clinical N-classification (*p* = 0.0027) as independent negative predictors of RFS, while high maximum SUVmax (*p* = 0.037), advanced clinical T-classification (*p* = 0.030), and advanced TNM stage (*p* = 0.0067) were independent negative predictors of OS. For recurrence and death in HER2-positive patients, high total TLG (*p* = 0.037, *p* = 0.0048, respectively) and advanced TNM stage (*p* = 0.048, *p* = 0.046, respectively) were independent prediction factors. In the triple-negative group, independent factors related to recurrence and death were high maximum SUVmax (*p* = 0.0014, *p* = 0.0003, respectively) and advanced TNM stage (*p* < 0.0001, *p* < 0.0001, respectively).

Materials and Methods: Records of 546 stage I–III invasive breast cancer patients, including 344 estrogen receptor (ER)-positive/human epidermal growth factor receptor 2 (HER2)-negative, 110 HER2-positive, and 92 triple-negative cases, treated at four institutions were reviewed retrospectively. Harmonized primary tumor and nodal maximum SUVmax, metabolic tumor volume (MTV), and TLG indicated in pretreatment FDG-PET/CT results were analyzed. Evaluations of relationships of clinicopathological factors, volume-based quantitative parameters, recurrence-free survival (RFS), and overall survival (OS) for each subtype were performed with a Cox proportional hazards model and log-rank test.

Conclusions: The results indicated that potential surrogate markers for prognosis in patients with the three main subtypes of operable breast cancer include harmonized pretreatment quantitative volume-based FDG-PET/CT parameters, particularly whole-lesion SUVmax and TLG.

## INTRODUCTION

Breast cancer is a heterogeneous group of diseases that show various types of histological differentiation and clinical courses, as well as responses to treatment. Management of affected patients is generally decided following examinations of obtained specimens to determine tumor-node-metastasis (TNM) stage, histologic tumor grade, and levels of hormone receptors and molecular markers [1]. Among prognosis-related immunohistochemical factors, including hormone receptors such as estrogen receptor (ER), progesterone receptor (PR), human epidermal growth factor receptor 2 (HER2), and Ki-67, ER-positive/HER2-negative, HER2-positive, and triple-negative, are three primary entities typically considered clinically based on immunohistochemical findings of the primary tumor biopsy specimen. Recently, noninvasive diagnostic tools have been gaining popularity for prediction of tumor behavior, with magnetic resonance spectroscopy (MRS) and diffusion-weighted imaging (DWI) with magnetic resonance imaging (MRI) reported to provide surrogate imaging biomarkers showing correlations with clinicopathological prognostic factors [2, 3]. In addition, standardized uptake value (SUV), metabolic tumor volume (MTV), and tumor lesion glycolysis (TLG) derived from ^18^F-fluorodeoxyglucose positron emission tomography/computed tomography (FDG-PET/CT) findings are reported to have correlations with clinicopathological prognostic factors and considered to be prognosis prediction factors [2–6].

Biological and technical factors related to the PET scanner model used, as well as the acquisition protocol and reconstruction algorithm, along with various parameters can result in varied SUV findings. Thus, a harmonization strategy is necessary for comparing semi-quantitative PET parameters among available imaging methods, which is a notably relevant issue for multicenter trials employing different PET systems. Established harmonization programs include the EANM/EARL program [7, 8] and Quantitative Imaging Biomarker Alliance (QIBA/UPICT) [9, 10], which provide comparisons of SUV metrics across different systems. Those are especially useful for multicenter studies, though can also be used by single institutions that may have several different PET systems. To the best of our knowledge, no previous study has evaluated harmonization of FDG-PET/CT quantitative parameters in breast cancer cases as potential surrogate markers. This investigation examined harmonized quantitative volume-based FDG-PET/CT parameters for predicting prognosis in patients with breast cancer.

## RESULTS

### Harmonization

**Figure 1:**
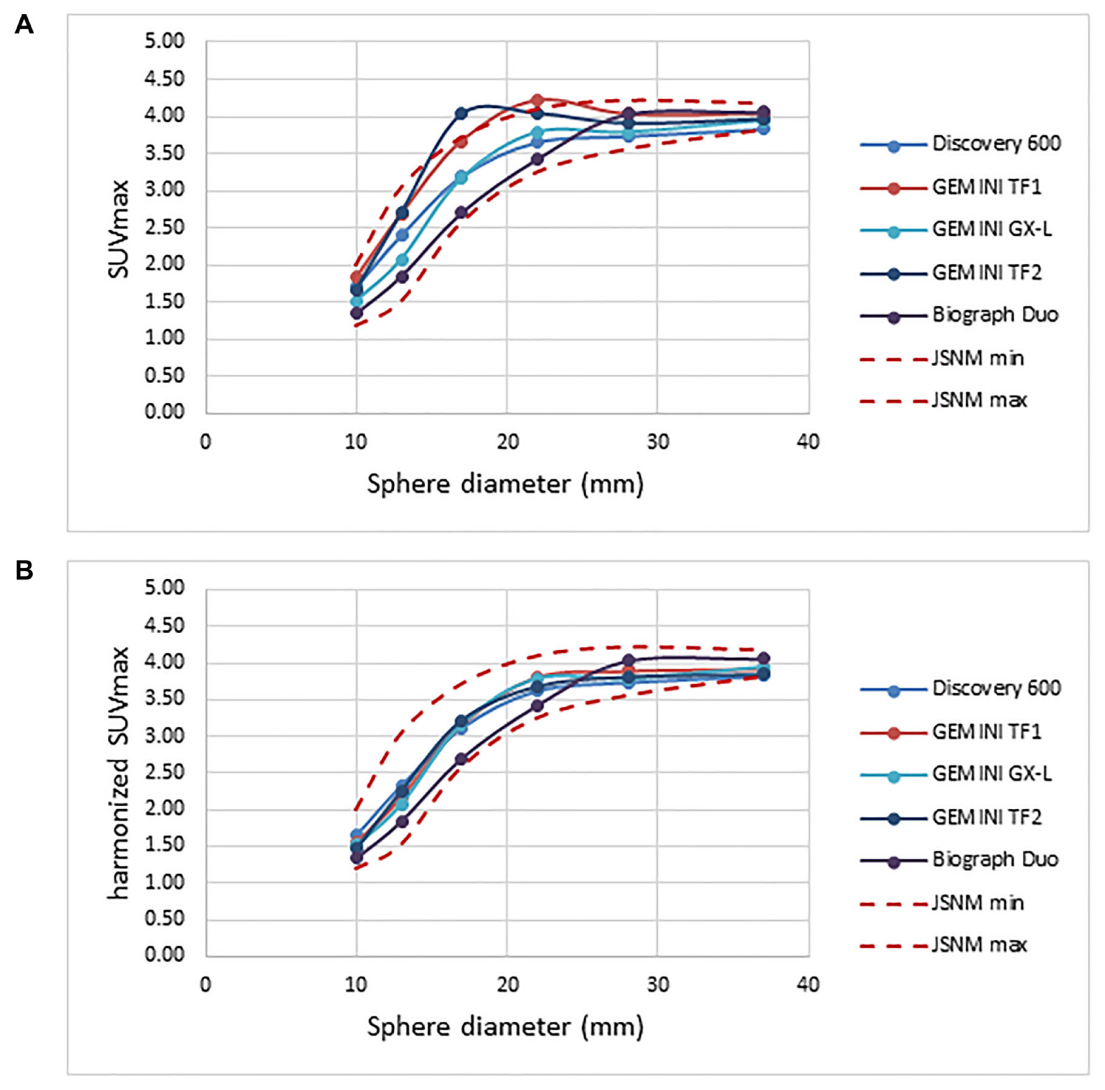
Recovery coefficient for five different PET scanners obtained from 30-minute PET imaging. (**A**) Pre-harmonization compared to (**B**) post-harmonization recovery coefficient.

With the GXL and Biograph Duo (no filter needed for either system), SUVmax fell within the JSNM reference range. The value for full-width at half maximum (FWHM) for the additional gaussian filter resulting in SUVmax within the JSNM reference was 3.4 mm for the Discovery 600, and 5.9 and 5.8 mm for the Gemini TF1 and TF2 systems, respectively (Figure 1).

### Patient characteristics

Table 1 shows patient and tumor characteristics. After excluding invasive ductal carcinoma, histology results for the ER-positive/HER2-negative type patients were myxoid carcinoma (*n* = 14), invasive lobular carcinoma (*n* = 5), and apocrine carcinoma (*n* = 1), while those in the HER2-positive type were myxoid carcinoma (*n* = 3), invasive lobular carcinoma (*n* = 2), and apocrine carcinoma (*n* = 2), and in triple-negative type invasive lobular carcinoma (*n* = 1) and apocrine carcinoma (*n* = 3).

**Table 1:** Patient and tumor characteristics

	ER-positive/HER2-negative	HER2-positive	Triple-negative
Number of patients	344	110	92
Age (years, mean ± SD)	58.7 ± 13.5	57.2 ± 13.6	56.5 ± 15.0
Histology			
IDC	324 (94.2%)	103 (93.6%)	88 (95.7%)
Others	20 (5.8%)	7 (6.4%)	4 (4.3%)
Primary longest size (mm, mean ± SD)	24.7 ± 13.9	33.6 ± 18.2	28.3 ± 13.7
Receptor positivity			
Estrogen receptor	324 (100%)	62 (56.4%)	0 (0%)
Progesterone receptor	285 (82.8%)	43 (39.1%)	0 (0%)
HER-2/neu	0 (0%)	110 (100%)	0 (0%)
Ki-67 index status			
< 20%	206 (59.9%)	22 (20.0%)	20 (21.7%)
≥ 20%	138 (40.1%)	88 (80.0%)	72 (78.3%)
Nuclear grade			
Grade1	241 (70.1%)	32 (29.1%)	24 (26.1%)
Grade2	64 (18.6%)	24 (21.8%)	28 (30.4%)
Grade3	39 (11.3%)	54 (49.1%)	40 (43.5%)
Clinical T status			
T1	164 (47.7%)	21 (19.1%)	28 (30.4%)
T2	153 (44.5%)	64 (58.2%)	55 (59.8%)
T3	15 (4.4%)	12 (10.9%)	2 (2.2%)
T4	12 (3.5%)	13 (11.8%)	7 (7.6%)
Pathological N status			
N0	212 (61.6%)	44 (40.0%)	44 (47.8%)
N1	110 (32.0%)	42 (38.2%)	38 (41.3%)
N2	12 (3.5%)	11 (10.0%)	5 (5.4%)
N3	10 (2.9%)	13 (11.8%)	5 (5.4%)
TNM Stage			
I	128 (37.2%)	15 (13.6%)	18 (19.6%)
II	173 (50.3%)	58 (52.7%)	57 (62.0%)
III	43 (12.5%)	37 (33.6%)	17 (18.5%)
NAC			
No	166 (48.3%)	41 (37.3%)	30 (32.6%)
Yes	178 (51.7%)	69 (62.7%)	62 (67.4%)

Data in parentheses are percentage. Abbreviations: SD: standard deviation, IDC: invasive ductal cancer, HER: human epidermal growth factor receptor, TNM: tumor-node-metastasis, NAC: neoadjuvant chemotherapy.

### ER-positive/HER2-negative type (*n* = 344)

There were 132 patients with pathological regional nodal metastasis, of whom 83 (62.9%) had abnormal FDG uptake in a regional node detected by FDG-PET/CT. Among those, primary tumor SUVmax was greater than nodal SUVmax in 64 (77.1%) and lower than nodal SUVmax in 19 (22.9%).

### OS analysis

**Figure 2:**
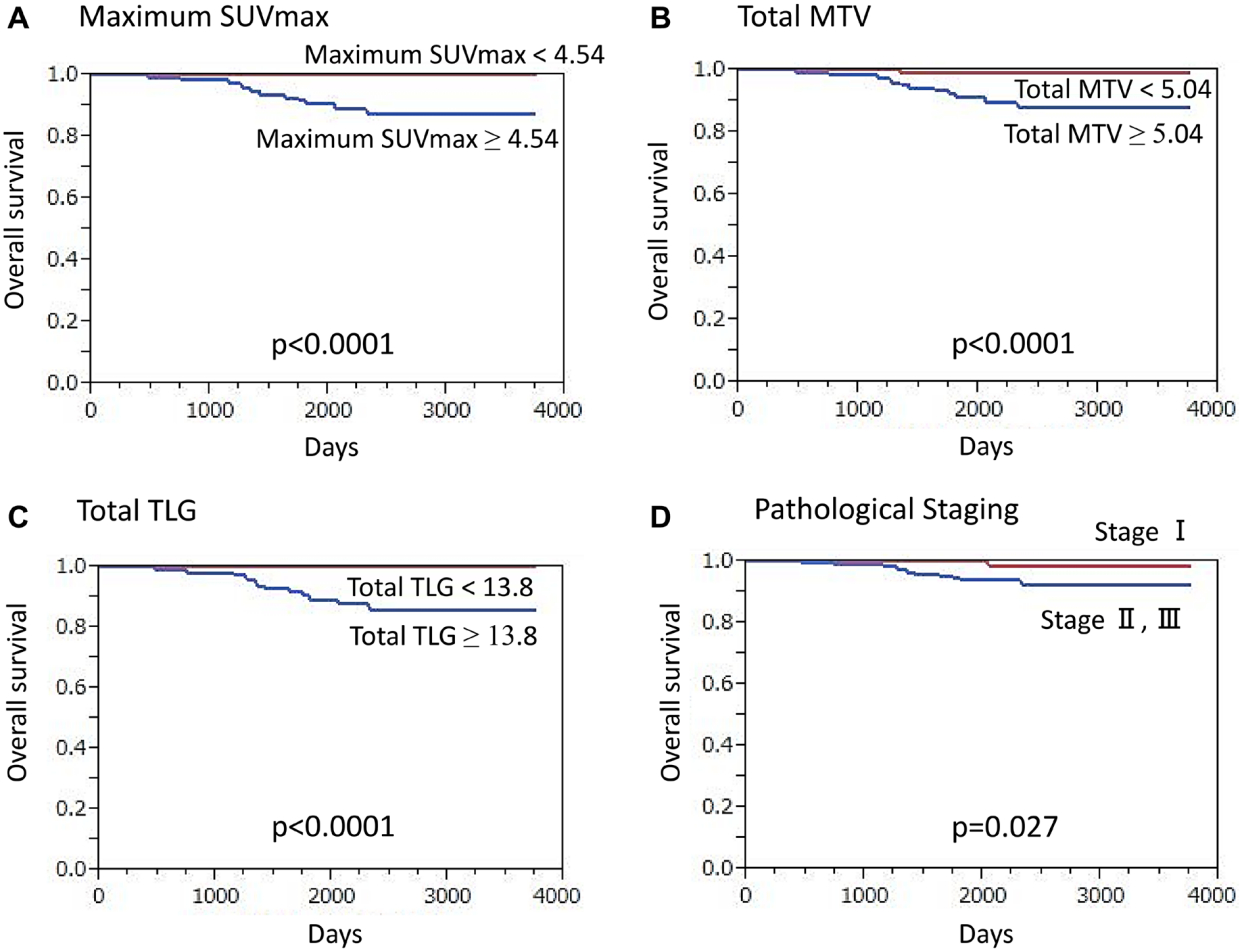
Overall survival (OS) of ER-positive/HER2-negative breast cancer patients (*n* = 344) (Kaplan-Meier survival analysis). A significantly lower OS rate was found in patients (**A**) with a high maximum SUVmax value (≥ 4.54) as compared to those with lower values (*p* < 0.0001), (**B**) with high total MTV (≥ 5.04) as compared to those with lower values (*p* < 0.0001), (**C**) with high total TLG (≥ 13.8) as compared to those with lower values (*p* < 0.0001), and (**D**) classified as stage II or III as compared to those classified as stage I (*p* = 0.027).

For the ER+/HER2 cancer patients, median overall follow-up duration was 70.5 months (13.9–125.6 months), during which 13 (3.8%) died. Patients who died from cancer had a significantly higher maximum SUVmax as compared to those who survived (11.33 ± 5.43 vs. 4.33 ± 3.02, *p* < 0.0001). ROC curve analysis and log-rank testing results showed a significantly lower OS rate for patients with high maximum SUVmax (≥ 4.54) as compared to a lower value (*p* < 0.0001) (Figure 2A). Furthermore, patients with death from cancer had a significantly higher total MTV that those who survived (53.13 ± 56.97 vs. 5.85 ± 7.53, *p* < 0.0001) and those with a high total MTV (≥ 5.04) had a significantly lower OS rate that those with a low total MTV (*p* < 0.0001) (Figure 2B). In addition, significantly higher TLG was seen in patients who had a cancer-related death than in survivors (401.37 ± 594.14 vs. 18.08 ± 33.57, *p* < 0.0001), while a significantly lower OS rate was noted in those with high total TLG (≥ 13.80) than in patients with low total TLG (*p* < 0.0001) (Figure 2C).

High maximum SUVmax (*p* < 0.0001), high total MTV (*p* < 0.0001), high total TLG (*p* < 0.0001), presence of clinical regional node metastasis (*p* < 0.0001), advanced clinical T classification (*p* = 0.0013), presence of pathological regional node metastasis (*p* < 0.0001), advanced TNM stage (*p* = 0.027) (Figure 2D), high Ki-67 expression (*p* = 0.0040), and presence of NAC (*p* = 0.014) were shown by univariate analysis to have a significant association with death from cancer (Supplementary Table 1). On the other hand, no such association was seen with histology subtype (*p* = 0.58), high nuclear grade (*p* = 0.34), ER-negative (*p* = 0.93), or PR-negative (*p* = 0.66). High maximum SUVmax (*p* = 0.037), advanced clinical T classification (*p* = 0.030), and advanced TNM stage (*p* = 0.00067) were revealed to be independent factors for death prediction in multivariate analysis.

### RFS analysis

Thirty-six (10.5%) ER+/HER2- cancer patients had recurrence, with a median overall follow-up period of 67.6 months (range 13.1–125.6 months). High maximum SUVmax (*p* < 0.0001), high total MTV (*p* < 0.0001), high total TLG (*p* < 0.0001), presence of clinical regional node metastasis (*p* < 0.0001), advanced clinical T classification (*p* < 0.0001), presence of pathological regional node metastasis (*p* < 0.0001), advanced TNM stage (*p* < 0.0001), high Ki-67 expression (*p* = 0.0098), and presence of NAC (*p* = 0.0043) were each shown by univariate analysis results to have a significant association with recurrence (Supplementary Table 1), whereas histology subtype (*p* = 0.65), high nuclear grade (*p* = 0.24), ER-negative (*p* = 0.79), and PR-negative (*p* = 0.18) were not significantly associated. High maximum SUVmax (*p* = 0.018), high total TLG (*p* = 0.010), and presence of regional node metastasis shown by PET (*p* = 0.0027) were independent factors for predicting recurrence in multivariate analysis results.

### HER2-positive type (*n* = 110)

Pathological regional nodal metastasis was noted in 66 patients and FDG-PET/CT results showed abnormal FDG uptake in a regional node in 49 (74.2%) of those cases, among which primary tumor SUVmax was higher than nodal SUVmax in 44 (89.8%).

#### OS analysis

**Figure 3:**
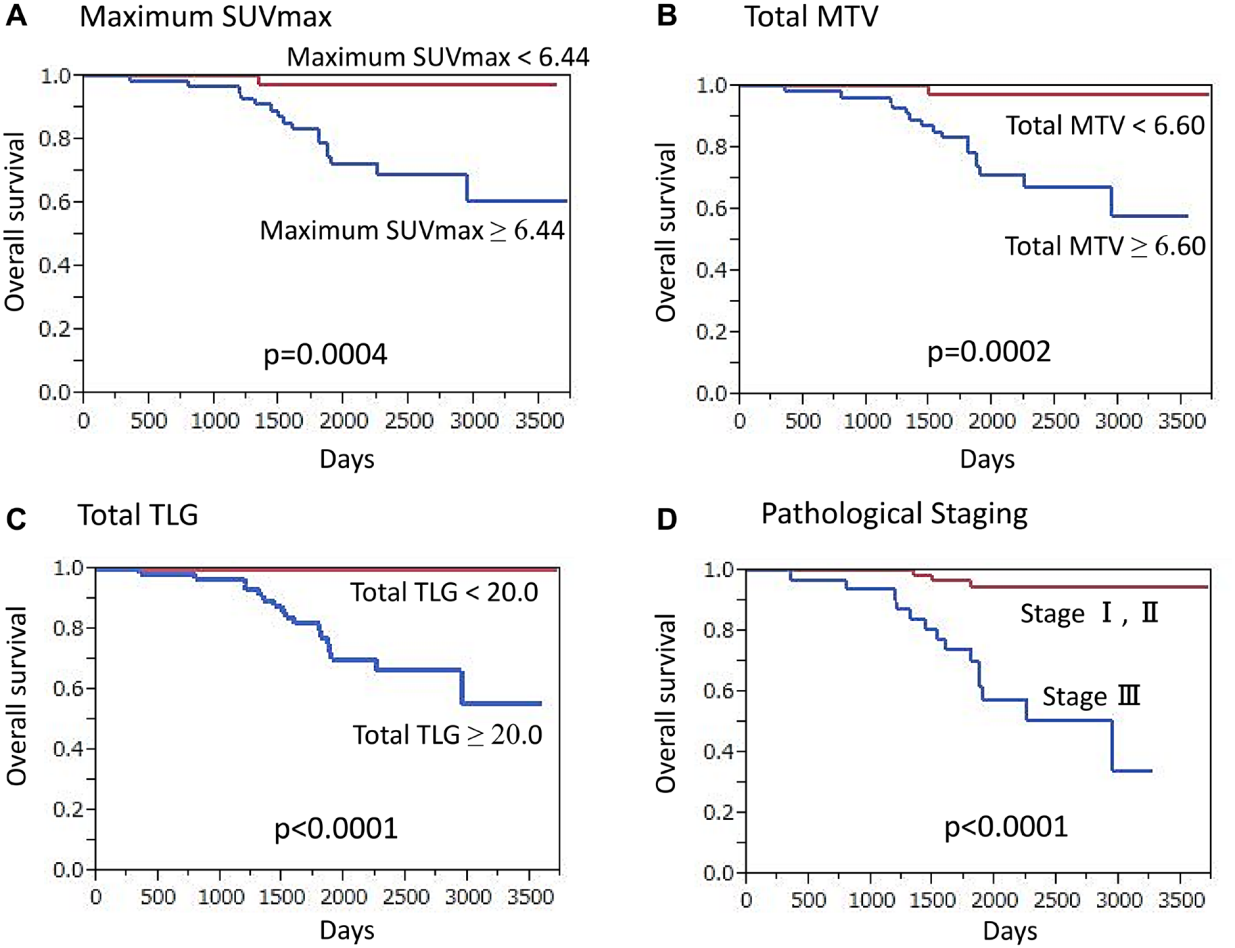
Overall survival (OS) of HER2-positive breast cancer patients (*n* = 110) (Kaplan-Meier survival analysis). A significantly lower OS rate was found in patients (**A**) with high maximum SUVmax (≥ 6.44) as compared to those with lower values (*p* = 0.0004), (**B**) with high total MTV (≥ 6.60) as compared to those with lower values (*p* = 0.0002), (**C**) with high total TLG (≥ 20.0) as compared to those with lower values (*p* < 0.0001), and (**D**) classified as stage III as compared to those classified as stage I or II (*p* < 0.0001).

HER2-positive cancer patients had a median overall follow-up period of 64.8 months (11.3–123.9 months), during which 18 (16.4%) died. A significantly higher maximum SUVmax was shown for those who died of cancer as compared to surviving patients (11.33 ± 5.43 vs. 4.33 ± 3.02, *p* < 0.0001). Also, a significantly lower OS rate was seen in patients with a high maximum SUVmax (≥ 6.44) as compared to a low maximum SUVmax (*p* = 0.0004) (Figure 3A). Additionally, patients with cancer death had a significantly higher total MTV than those who survived (49.99 ± 37.32 vs. 13.15 ± 23.13, *p* < 0.0001), while patients with high total MTV (≥ 6.60) had a significantly lower OS rate that those with low total MTV (*p* = 0.0002) (Figure 3B). In cancer death cases, significantly higher TLG as compared to survival cases was found (285.43 ± 255.62 vs. 66.68 ± 166.76, *p* < 0.0001) and patients with high total TLG (≥ 20.0) had a significantly lower OS rate than those with low total TLG (*p* < 0.0001) (Figure 3C).

Univariate analysis results demonstrated that high maximum SUVmax (*p* = 0.0004), high total MTV (*p* = 0.0002), high total TLG (*p* < 0.0001), presence of clinical regional node metastasis (*p* = 0.0003), advanced clinical T classification (*p* < 0.0001), presence of pathological regional node metastasis (*p* = 0.0008), advanced TNM stage (*p* < 0.0001) (Figure 3D), high nuclear grade (*p* = 0.011), and high Ki-67 expression (*p* = 0.049) were significantly associated with death (Supplementary Table 2). High total TLG (*p* = 0.0048) and advanced TNM stage (*p* = 0.046) were independent factors for predicting death in multivariate analysis.

#### RFS analysis

A median overall follow-up period of 59.6 months (8.3–123.9 months) was found in the HER2-positive cancer patients, of whom 35 (31.8%) demonstrated recurrence. Univariate analysis results showed that high maximum SUVmax (*p* < 0.0001), high total MTV (*p* < 0.0001), high total TLG (*p* < 0.0001), presence of clinical regional node metastasis (*p* < 0.0001), advanced clinical T classification (*p* < 0.0001), presence of pathological regional node metastasis (*p* < 0.0001), advanced TNM stage (*p* < 0.0001), high nuclear grade (*p* = 0.0014), and high Ki-67 expression (*p* = 0.0027) had a significant association with recurrence (Supplementary Table 2). In results of multivariate analysis, high total TLG (*p* = 0.037) and advanced TNM stage (*p* = 0.024) were found to be independent factors for recurrence prediction.

### Triple-negative (*n* = 92)

Among the 48 patients with pathological regional nodal metastasis, abnormal FDG uptake in a regional node was detected in 42 (87.5%) by FDG-PET/CT. Of those 42, primary tumor SUVmax was higher than nodal SUVmax in 29 (69.0%).

#### OS analysis

**Figure 4:**
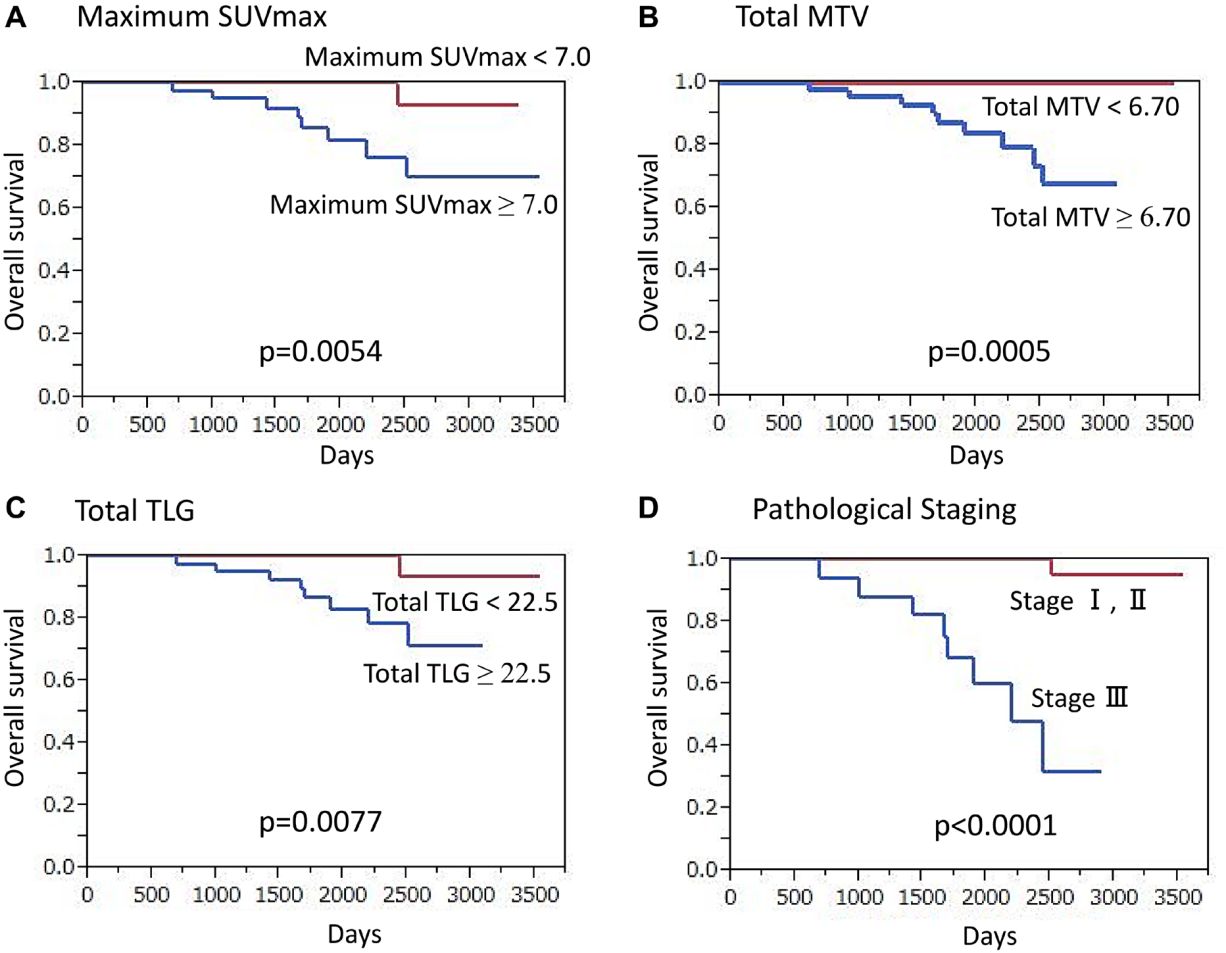
Overall survival (OS) of triple-negative breast cancer patients (*n* = 92) (Kaplan-Meier survival analysis). A significantly lower OS rate was found in patients (**A**) with high maximum SUVmax (≥ 7.0) as compared to those with lower values (*p* = 0.0054), (**B**) with high total MTV (≥ 6.7) as compared to those with lower values (*p* = 0.0005), (**C**) with high total TLG (≥ 22.5) as compared to those with lower values (*p* = 0.0077), and (**D**) classified as stage III as compared to those classified as stage I or II (*p* < 0.0001).

For triple negative cancer patients, median overall follow-up was 64.8 months (15.0–117.9 months), during which 9 (9.8%) died. Those who died of cancer had a significantly higher maximum SUVmax as compared to patients who survived (10.91 ± 4.14 vs. 6.75 ± 4.25, *p* = 0.0064), while those with a high maximum SUVmax (≥ 7.0) had a significantly lower OS rate as compared to patients with a low maximum SUVmax (*p* = 0.0054) (Figure 4A). Additionally, a significantly higher total MTV was noted in cancer death than survival cases (35.28 ± 44.22 vs. 8.31 ± 9.14, *p* < 0.0001) and patients with high total MTV (≥ 6.70) showed a significantly lower OS rate as compared to those with low total MTV (*p* = 0.0005) (Figure 4B). Cancer death cases also showed significantly higher TLG as compared to survival cases (258.7 ± 448.2 vs. 40.03 ± 53.8, *p* < 0.0001) and patients with high total TLG (≥ 22.5) had a significantly lower OS rate as compared to those with low total TLG (*p* = 0.0077) (Figure 4C).

Univariate analysis revealed that high maximum SUVmax (*p* = 0.0054), high total MTV (*p* = 0.0005), high total TLG (*p* = 0.0077), presence of clinical regional node metastasis (*p* = 0.0001), advanced clinical T classification (*p* = 0.0017), presence of pathological regional node metastasis (*p* = 0.0072), and advanced TNM stage (*p* < 0.0001) (Figure 4D) had a significant association with death (Supplementary Table 3). In multivariate analysis, results showed high maximum SUVmax (*p* = 0.0003) and advanced TNM stage (*p* < 0.0001) as independent factors for predicting death.

#### RFS analysis

For triple negative cancer patients, the median overall follow-up period was 59.4 months (12.5–117.9 months), during which time 20 (21.7%) had recurrence. Univariate analysis results revealed that high maximum SUVmax (*p* = 0.0021), high total MTV (*p* = 0.0003), high total TLG (*p* = 0.0036), presence of clinical regional node metastasis (*p* = 0.014), advanced clinical T classification (*p* = 0.0021), presence of pathological regional node metastasis (*p* = 0.016), advanced TNM stage (*p* < 0.0001), and presence of NAC (*p* = 0.0047) were significantly associated with recurrence (Supplementary Table 3), while high maximum SUVmax (*p* = 0.0014), advanced TNM stage (*p* < 0.0001), and presence of NAC (*p* = 0.0037) were shown in multivariate analysis results to be independent factors for recurrence prediction.

## DISCUSSION

This is the first known investigation of breast cancer patients (stage I–III) that used harmonized pretreatment quantitative volume-based FDG-PET/CT parameters for prognosis prediction. Primary tumor and nodal SUVmax, as well as TLG values were found to be prominent potential surrogate markers for prognosis in patients with the three major subtypes, indicating their importance for providing better individualized care.

Establishment of different PET/CT scanner types at the same institution is becoming common, which affects both clinical practice and multicenter trials. Thus, methods for harmonization of PET quantitative values are needed. There have been several evaluations of the utility of SUVmax harmonization for evaluating response to chemotherapy in various types of cancer patients, including lung, cervical, and rectal, as well as colorectal liver metastasis, malignant lymphoma, and malignant melanoma. Those findings have demonstrated a significant association of harmonized metabolic PET response classification with final clinical response assessment and its better ability for predicting disease-free survival as compared to a non-harmonized PET classification [11–14]. Another recent study also presented findings showing harmonized SUVmax to be an independent prognostic factor of progression-free survival in non-small-cell lung cancer patients [15]. PET used for texture analysis or radiomic studies is sensitive to acquisition and reconstruction parameters, which can hinder pooling of data acquired with different scanners or protocols. Efficient harmonization methods for multicenter radiomic studies of patients with breast and lung cancer that use FDG-PET/CT have been proposed by two different groups [16, 17].

As for tumor subtype in the present cohort, the mean maximum SUVmax values for HER2-positive and triple-negative tumors were 7.92 ± 4.59 and 7.16 ± 4.40, respectively, both higher than that for ER-positive/HER2-negative tumors (4.59 ± 3.40) (*p* < 0.001 and *p* < 0.001, respectively), findings similar to previous reports [4, 6, 18]. Other studies of breast cancer have found that primary tumor quantitative FDG-PET/CT parameters are correlated with known prognostic pathological parameters, while those were also shown useful for prognosis prediction without the need for division according to molecular subtype [2, 4–6]. More recently, reports regarding the usefulness of volume-based quantitative parameters (SUVmax, MTV, TLG) for predicting prognosis using major receptor types have been presented. A study of ER+/HER2- cancer patients showed primary tumor MTV as an independent factor for predicting recurrence [19], while primary tumor SUVmax was demonstrated to be an independent factor in another report [20]. Also, primary tumor SUVmax was shown to be an independent factor with influence on recurrence in triple-negative cancer patients [21]. The present results are similar to those, though this is the first known evaluation HER2-positive cancer cases to be presented.

Although previous studies of stage operable breast cancer patients only evaluated primary tumor quantitative FDG-PET/CT values as imaging biomarkers [2, 4–6, 18–21], cases with a lower primary tumor SUVmax value as compared to that for nodal SUVmax are commonly encountered in clinical situations. In the present cohort as well, it was observed in 10.2–30.9% and we defined that as maximum SUVmax, which is considered to be a strong point of this report.

The present study has limitations including its retrospective design, as that may limit generalization of the conclusions and could lead to statistical errors. Furthermore, in the enrolled patients, heterogeneous chemotherapy and hormonal therapy regimens were used.

## MATERIALS AND METHODS

### Patient recruitment

This retrospective study of FDG-PET/CT findings was conducted by Hyogo College of Medicine Hospital, Kindai University Hospital, Nippon Medical School Hospital, and National Cancer Center Hospital. An appropriate review board at each gave approval to the protocol and waived the need for patient-informed consent. A total of 606 records of females newly diagnosed with stage I-III invasive breast cancer and who underwent pretreatment FDG-PET/CT examinations between January 2010 and December 2016 were examined. Exclusion criteria were bilateral breast cancer (*n* = 15), no focal uptake shown by FDG-PET/CT (*n* = 24), invasive tumor size < 1 cm (*n* = 13), and no immunohistochemical data available (*n* = 8), thus 546 patients were finally enrolled. A mastectomy was performed in 315 and 231 underwent breast-conserving surgery. Tumor subtype was ER-positive/HER2-negative in 344, HER2-positive in 110, and triple-negative in 92 of the patients. To determine T status, magnetic resonance imaging (MRI) and ultrasound, and also clinical examination results were employed. Axillary node diagnostic tool determination was based on aspiration cytology, sentinel node biopsy, and axillary node dissection assessments, while for histopathologic characteristics, core needle biopsy and surgical resection findings were used.

Of the total cohort, 178 ER-positive/HER2-negative breast cancer, 69 HER2-positive cancer, and 62 triple-negative patients underwent neoadjuvant chemotherapy (NAC) and/or hormonal therapy prior to surgery. For NAC, an anthracycline-containing regimen, anthracycline followed by taxane, or taxane-based regimen was administered. Hormonal therapy was administered to patients with hormone receptor-positive cancer and a trastuzumab-based regimen to those with HER2-positive.

Adjuvant chemotherapy or hormonal therapy was administered, and radiotherapy was also performed after surgery in all cases. Mammography, ultrasound, CT, bone scanning, and FDG-PET/CT were used for determining disease recurrence, metastasis, and progression during follow-up. Any suggestive lesion was histologically confirmed by fine-needle aspiration cytology or clinical follow-up findings, including therapeutic response.

### FDG-PET/CT

The participating institutions employed different PET/CT scanner systems, including Gemini GXL (Philips Medical Systems, Eindhoven, The Netherlands), Gemini TF (Philips Medical Systems), Biograph Duo (Siemens Healthcare, Erlangen, Germany), and Discovery 600 (GE Healthcare, WI, USA), and their clinical parameters are presented in Table 2. Prior to the examination, patients fasted for five hours and blood glucose was measured immediately prior to injection of FDG at 3.0–4.0 MBq/kg of body weight. None had a blood glucose level greater than 200 mg/dL. Approximately 60 minutes after the injection, static emission images were obtained, during which the patients were allowed to breathe normally.

**Table 2:** Clinical parameters of PET scanners

Scanner	Gemini GXL	Gemini TF1	Gemini TF2	Biograph Duo	Discovery 600
Vender	Philips	Philips	Philips	SIEMENS	GE
PET scanning					
FDG injection dose (MBq/kg)	4	3	4	3	4
Scan time (sec) for each bed	90	90	80	110	120
TOF	no	yes	yes	no	no
PET reconstruction					
Reconstruction	line-of-response row-action maximum likelihood algorithm (LOR-RAMLA)	3D-OSEM	Full-list mode TOF 3D-OSEM	3D-OSEM	3D-OSEM
Iterations	2	3	3	2	2
Subsets	n/a	33	33	8	16
Smoothing	n/a	n/a	n/a	Gaussian	Gaussian
FWHM of filter (mm)				5	5
Matrix	144 × 144	144 × 144	144 × 144	128 × 128	192 × 192
Pixel size (mm)	4 × 4 × 4	4 × 4 × 4	4 × 4 × 4	5.31 × 5.31 × 5	2.6 × 2.6 × 2.6
PSF	no	no	no	no	no

Abbreviations: FDG: fluorodeoxyglucose, TOF: time of flight, OSEM: ordered-subset expectation maximization, FWHM: full-width at half maximum, PSF: point spread function.

An experienced reader (12 years of experience with oncologic FDG-PET/CT) who had no knowledge of other imaging results or clinical and histopathologic data retrospectively reviewed all of the FDG-PET/CT images. RAVAT (Nihon Medi-Physics Co. Ltd., Tokyo, Japan), a commercially available software package able to harmonize SUVs obtained with different PET/CT systems in a range advocated by the Japanese Society of Nuclear Medicine (JSNM) using phantom data, was employed [22]. SUVmax was defined as maximum SUV within the target volume and determined using the following formula: concentration of radioactivity in volume of interest (VOI) (MBq/ml) × total body weight (kg)/injected radioactivity (g/MBq). As for SUVmean, that was calculated as summed SUV in each voxel in the target volume divided by the number of voxels within the target volume. MTV was measured automatically inside the tumor VOI, with the margin threshold set at 40% of SUVmax. Then, TLG was t as SUVmean × MTV, taking into consideration both metabolic activity and tumor burden. “Maximum SUVmax” was defined as the maximum for all lesions (primary tumor and regional nodal metastases), while “total MTV” and “total TLG” were calculated by summing the corresponding values for each lesion in individual patients.

### Histological analysis

ER, PR, HER2, and Ki67 expression levels were examined in tissue samples that had undergone immunohistochemical staining, as well as formalin fixation and paraffin embedding, with quantitative levels determined. In addition, the percentage of nuclear staining in cancer cells for ER, PR, and Ki67 was determined, with the cutoff value for ER and PR set at 1%, and that for Ki67 at 20%. Tumors that had an immunohistochemical score of 3 or showed *in situ* hybridization-positive fluorescence with an immunohistochemical score of 2 were defined as HER2-positive.

### Statistical analysis

Student’s *t* test was used for comparisons of patients with or without recurrence or death, and to determine the optimal threshold receiver operating characteristics (ROC) analysis was performed. Recurrence-free survival (RFS), defined as time elapsed from date of diagnosis to recurrence, and overall survival (OS), time until death or date of the last follow-up visit when neither progression nor death occurred during follow-up were evaluated in this long-term follow-up study. The Kaplan-Meier method and log-rank test results were employed to determine survival curves.

Cox proportional hazards logistic regression was used to evaluate the prognostic value of individual variables. To quantify risk of recurrence and death for primary clinical T classification, ER, PR, HER2, Ki-67, nuclear grade, regional node metastasis shown by PET (clinical N classification), pathological regional node metastasis (pathological N classification), TNM stage, histology subtype, maximum SUVmax, total MTV, and total TLG, univariate Cox proportional hazards modeling was used. Then, significant or borderline univariate variables (*p* < 0.1) were subjected to multivariate analysis, except for parameters with insufficient data. Cox model results are expressed as hazard ratio with 95% confidence interval (CI). The SAS software package, version 9.3 (SAS Institute Inc., Cary, NC, USA), was used for statistical analysis, with *p* < 0.05 considered to indicate significance.

### Ethical approval

This retrospective study was approved by appropriate institutional review boards and the need for patient informed consent was waived.

## CONCLUSIONS

We concluded that harmonized quantitative volume-based values, especially those for the primary tumor and nodal SUVmax and TLG, obtained with FDG-PET/CT can provide useful information regarding prognosis for both recurrence and death in patients with operable invasive breast cancer, including all three main subtypes. The findings presented here are considered useful for improving care of individual patients.

## SUPPLEMENTARY MATERIALS









## Abbreviations

ER: estrogen receptor
HER2: human epidermal growth factor receptor 2
SUV: standardized uptake value
MTV: metabolic tumor volume
TLG: total lesion glycolysis
RFS: recurrence-free survival
OS: overall survival
TNM: tumor-node-metastasis
PR: progesterone receptor
FDG: ^18^F-fulorodeoxyglucose
PET/CT: positron emission tomography/computed tomography
MRI: magnetic resonance imaging
NAC: neoadjuvant chemotherapy
LOR-RAMLA: line-of-response row-action maximum likelihood algorithm
OSEM: ordered-subset expectation maximization
TOF: time of flight
JSNM: Japanese Society of Nuclear Medicine
VOI: volume of interest
ROC: receiver operating characteristics
CI: confidence interval
