# Supplementary material for: Harmonized pretreatment quantitative volume-based FDG-PET/CT parameters for prognosis of stage I–III breast cancer: Multicenter study
Source: Oncotarget. 2021 Jan 19;12(2):95–105. doi: 10.18632/oncotarget.27851 (PMC7825640; doi:10.18632/oncotarget.27851)
Supplement: Supplementary file 1 [file oncotarget-12-95-s001.pdf]

Harmonized pretreatment quantitative volume-based FDG-PET/CT parameters for prognosis of stage I–III breast cancer: Multicenter study

SUPPLEMENTARY MATERIALS

Supplementary Table 1: Univariate and multivariate analysis of PFS and OS in 344 patients with ER-positive/HER2-negative breast cancer. See Supplementary Table 1

Supplementary Table 2: Univariate and multivariate analysis of PFS and OS in 110 patients with HER2-positive breast cancer. See Supplementary Table 2

Supplementary Table 3: Univariate and multivariate analysis of PFS and OS in 92 patients with triple-negative breast cancer. See Supplementary Table 3
