# Supplementary material for: Harmonized pretreatment quantitative volume-based FDG-PET/CT parameters for prognosis of stage I–III breast cancer: Multicenter study
Source: Oncotarget. 2021 Jan 19;12(2):95–105. doi: 10.18632/oncotarget.27851 (PMC7825640; doi:10.18632/oncotarget.27851)
Supplement: Supplementary file 2 [file oncotarget-12-95-s002.docx]

**Supplementary Table 1: Univariate and multivariate analysis of PFS and OS in 344 patients with ER-positive/HER2-negative breast cancer**

|  |  | **Progression free survival** | | | | **Overall survival** | | | |
| --- | --- | --- | --- | --- | --- | --- | --- | --- | --- |
|  |  | **Univariate analysis** | | **Multivariate analysis** | | **Univariate analysis** | | **Multivariate analysis** | |
| **Variable** | **N** | **p value** | **HR (95% CI)** | **p value** | **HR (95% CI)** | **p value** | **HR (95% CI)** | **p value** | **HR (95% CI)** |
| Highest SUVmax |  | <0.0001 |  | 0.018 |  | <0.0001 |  | 0.037 |  |
| < 4.54 | 212 |  | 1.00 |  | 1.00 |  | 1.00 |  | 1.00 |
| ≥4.54 | 132 |  | 3.91 (1.98-5.63) |  | 3.41 (1.88-7.19) |  | 4.47 (2.69-11.26) |  | 4.25 (2.31-8.14) |
| Total MTV (mL) |  | <0.0001 |  | 0.24 |  | <0.0001 |  | 0.88 |  |
| < 5.04 | 211 |  | 1.00 |  | 1.00 |  | 1.00 |  | 1.00 |
| ≥5.04 | 133 |  | 3.32 (1.79-5.27) |  | 1.18 (0.94-1.49) |  | 3.41 (1.79-5.02) |  | 1.15 (0.79-1.44) |
| Total TLG (g) |  | <0.0001 |  | 0.010 |  | <0.0001 |  | 0.14 |  |
| < 13.8 | 223 |  | 1.00 |  | 1.00 |  | 1.00 |  | 1.00 |
| ≥13.8 | 121 |  | 4.67 (2.09-8.23) |  | 3.87 (2.17-7.67) |  | 3.89 (2.32-6.52) |  | 2.17 (1.67-2.88) |
| PET N classification |  | <0.0001 |  | 0.0027 |  | <0.0001 |  | 0.053 |  |
| cN0 | 261 |  | 1.00 |  | 1.00 |  | 1.00 |  | 1.00 |
| cN1, N2, N3 | 83 |  | 3.52 (1.87-6.01) |  | 5.73 (2.16-12.38) |  | 4.38 (2.48-8.74) |  | 3.97 (2.19-7.64) |
| Clinical T classification |  | <0.0001 |  | 0.26 |  | 0.0013 |  | 0.030 |  |
| cT1 | 164 |  | 1.00 |  | 1.00 |  | 1.00 |  | 1.00 |
| cT2, T3, T4 | 180 |  | 3.41 (1.83-5.16) |  | 1.21 (0.83-1.52) |  | 4.45 (2.76-11.59) |  | 4.32 (2.34-8.55) |
| Pathological N classification |  | <0.0001 |  | 0.46 |  | <0.0001 |  | 0.59 |  |
| pN0 | 212 |  | 1.00 |  | 1.00 |  | 1.00 |  | 1.00 |
| pN1, N2, N3 | 132 |  | 3.47 (1.85-5.73) |  | 1.12 (0.74-1.56) |  | 3.64 (2.03-6.19) |  | 1.13 (0.83-1.57) |
| Pathological Stage |  | <0.0001 |  | 0.11 |  | 0.027 |  | 0.0067 |  |
| Ⅰ | 128 |  | 1.00 |  | 1.00 |  | 1.00 |  | 1.00 |
| Ⅱ, Ⅲ | 216 |  | 3.45 (1.88-5.89) |  | 1.41 (1.05-1.81) |  | 4.55 (3.08-10.79) |  | 5.75 (3.21-12.59) |
| Histology |  | 0.65 |  |  |  | 0.58 |  |  |  |
| Invasive ductal carcinoma | 324 |  | 1.00 |  |  |  | 1.00 |  |  |
| Others | 20 |  | 0.82 (0.57-1.31) |  |  |  | 0.92 (0.62-1.39) |  |  |
| Tumor grade |  | 0.24 |  |  |  | 0.34 |  |  |  |
| 1 | 241 |  | 1.00 |  |  |  | 1.00 |  |  |
| 2, 3 | 103 |  | 1.07 (0.75-1.45) |  |  |  | 1.05 (0.74-1.48) |  |  |
| Estrogen receptor status |  | 0.79 |  |  |  | 0.93 |  |  |  |
| Positive | 344 |  | 1.00 |  |  |  | 1.00 |  |  |
| Negative | 0 |  | 0.73 (0.57-1.27) |  |  |  | 0.69 (0.55-1.04) |  |  |
| Progesterone receptor status |  | 0.18 |  |  |  | 0.66 |  |  |  |
| Positive | 285 |  | 1.00 |  |  |  | 1.00 |  |  |
| Negative | 59 |  | 1.05 (0.79-1.47) |  |  |  | 0.89 (0.59-1.35) |  |  |
| Ki-67 expression level |  | 0.0098 |  | 0.76 |  | 0.0040 |  | 0.61 |  |
| <20% | 206 |  | 1.00 |  | 1.00 |  | 1.00 |  | 1.00 |
| ≥20% | 138 |  | 1.89 (1.23-2.68) |  | 0.96 (0.52-1.41) |  | 3.77 (2.12-5.11) |  | 1.05 (0.76-1.67) |
| NAC |  | 0.0043 |  | 0.94 |  | 0.014 |  | 0.71 |  |
| No | 166 |  | 1.00 |  | 1.00 |  | 1.00 |  | 1.00 |
| Yes | 178 |  | 1.98 (1.27-2.95) |  | 0.93 (0.48-1.37) |  | 1.68 (1.27-2.65) |  | 1.02 (0.68-1.56) |

Abbreviations: SUVmax: maximum standardized uptake value, MTV: metabolic tumor volume, TLG: total lesion glycolysis, PET: positron emission tomography, NAC: neoadjuvant chemotherapy, HR: hazard ratio, CI: confidence interval.
