# Supplementary material for: Harmonized pretreatment quantitative volume-based FDG-PET/CT parameters for prognosis of stage I–III breast cancer: Multicenter study
Source: Oncotarget. 2021 Jan 19;12(2):95–105. doi: 10.18632/oncotarget.27851 (PMC7825640; doi:10.18632/oncotarget.27851)
Supplement: Supplementary file 3 [file oncotarget-12-95-s003.docx]

**Supplementary Table 2: Univariate and multivariate analysis of PFS and OS in 110 patients with HER2-positive breast cancer**

|  |  | **Progression free survival** | | | | **Overall survival** | | | |
| --- | --- | --- | --- | --- | --- | --- | --- | --- | --- |
|  |  | **Univariate analysis** | | **Multivariate analysis** | | **Univariate analysis** | | **Multivariate analysis** | |
| **Variable** | **N** | **p value** | **HR (95% CI)** | **p value** | **HR (95% CI)** | **p value** | **HR (95% CI)** | **p value** | **HR (95% CI)** |
| Highest SUVmax |  | <0.0001 |  | 0.42 |  | 0.0004 |  | 0.46 |  |
| < 6.44 | 50 |  | 1.00 |  | 1.00 |  | 1.00 |  | 1.00 |
| ≥6.44 | 60 |  | 3.49 (1.87-5.76) |  | 1.01 (0.77-1.49) |  | 3.43 (2.03-5.78) |  | 1.11 (0.84-1.46) |
| Total MTV (mL) |  | <0.0001 |  | 0.85 |  | 0.0002 |  | 0.55 |  |
| < 6.60 | 49 |  | 1.00 |  | 1.00 |  | 1.00 |  | 1.00 |
| ≥6.60 | 61 |  | 3.32 (1.65-5.56) |  | 0.71 (0.48-1.43) |  | 3.55 (2.54-6.19) |  | 1.16 (0.88-1.49) |
| Total TLG (g) |  | <0.0001 |  | 0.037 |  | <0.0001 |  | 0.048 |  |
| < 20.0 | 48 |  | 1.00 |  | 1.00 |  | 1.00 |  | 1.00 |
| ≥20.0 | 62 |  | 5.25 (3.37-8.78) |  | 3.61 (2.39-5.12) |  | 4.11 (2.65-9.67) |  | 4.36 (2.48-6.44) |
| PET N classification |  | <0.0001 |  | 0.58 |  | 0.0003 |  | 0.33 |  |
| cN0 | 61 |  | 1.00 |  | 1.00 |  | 1.00 |  | 1.00 |
| cN1, N2, N3 | 49 |  | 3.42 (1.77-5.69) |  | 1.13 (0.87-1.46) |  | 3.48 (2.21-5.89) |  | 1.23 (0.87-1.74) |
| Clinical T classification |  | <0.0001 |  | 0.34 |  | <0.0001 |  | 0.058 |  |
| cT1, T2 | 85 |  | 1.00 |  | 1.00 |  | 1.00 |  | 1.00 |
| cT3, T4 | 25 |  | 3.50 (1.88-5.79) |  | 1.11 (0.86-1.47) |  | 4.03 (2.55-9.34) |  | 4.31 (2.32-6.28) |
| Pathological N classification |  | <0.0001 |  | 0.72 |  | 0.0008 |  | 0.53 |  |
| pN0 | 44 |  | 1.00 |  | 1.00 |  | 1.00 |  | 1.00 |
| pN1, N2, N3 | 66 |  | 3.42 (1.72-5.61) |  | 1.07 (0.81-1.42) |  | 3.36 (1.85-5.21) |  | 1.22 (0.81-1.79) |
| Pathological Stage |  | <0.0001 |  | 0.024 |  | <0.0001 |  | 0.046 |  |
| Ⅰ,Ⅱ | 73 |  | 1.00 |  | 1.00 |  | 1.00 |  | 1.00 |
| Ⅲ | 37 |  | 3.46 (1.81-5.87) |  | 3.87 (2.56-5.43) |  | 4.97 (3.47-11.75) |  | 4.24 (2.56-6.87) |
| Histology |  | 0.30 |  |  |  | 0.17 |  |  |  |
| Invasive ductal carcinoma | 103 |  | 1.00 |  |  |  | 1.00 |  |  |
| Others | 7 |  | 1.11 (0.75-1.43) |  |  |  | 1.19 (0.86-1.48) |  |  |
| Tumor grade |  | 0.0014 |  | 0.89 |  | 0.011 |  | 0.51 |  |
| 1, 2 | 56 |  | 1.00 |  | 1.00 |  | 1.00 |  | 1.00 |
| 3 | 54 |  | 2.27 (1.59-3.28) |  | 0.69 (0.49-1.37) |  | 1.72 (1.31-2.69) |  | 1.21 (0.86-1.51) |
| Estrogen or progesterone receptor status |  | 0.21 |  |  |  | 0.11 |  |  |  |
| Positive | 69 |  | 1.00 |  |  |  | 1.00 |  |  |
| Negative | 41 |  | 1.17 (0.85-1.45) |  |  |  | 1.19 (0.88-1.49) |  |  |
| Ki-67 expression level |  | 0.0027 |  | 0.19 |  | 0.049 |  | 0.85 |  |
| <20% | 22 |  | 1.00 |  | 1.00 |  | 1.00 |  | 1.00 |
| ≥20% | 88 |  | 2.22 (1.56-3.22) |  | 1.23 (1.02-1.57) |  | 1.54 (1.21-2.24) |  | 0.73 (0.51-1.41) |
| NAC |  | 0.27 |  |  |  | 0.20 |  |  |  |
| No | 41 |  | 1.00 |  |  |  | 1.00 |  |  |
| Yes | 69 |  | 1.15 (0.82-1.49) |  |  |  | 1.18 (0.86-1.48) |  |  |

Abbreviations: SUVmax: maximum standardized uptake value, MTV: metabolic tumor volume, TLG: total lesion glycolysis, PET: positron emission tomography, NAC: neoadjuvant chemotherapy, HR: hazard ratio, CI: confidence interval.
