# Supplementary material for: Harmonized pretreatment quantitative volume-based FDG-PET/CT parameters for prognosis of stage I–III breast cancer: Multicenter study
Source: Oncotarget. 2021 Jan 19;12(2):95–105. doi: 10.18632/oncotarget.27851 (PMC7825640; doi:10.18632/oncotarget.27851)
Supplement: Supplementary file 4 [file oncotarget-12-95-s004.docx]

**Supplementary Table 3: Univariate and multivariate analysis of PFS and OS in 92 patients with triple-negative breast cancer**

|  |  | **Progression free survival** | | | | **Overall survival** | | | |
| --- | --- | --- | --- | --- | --- | --- | --- | --- | --- |
|  |  | **Univariate analysis** | | **Multivariate analysis** | | **Univariate analysis** | | **Multivariate analysis** | |
| **Variable** | **N** | **p value** | **HR (95% CI)** | **p value** | **HR (95% CI)** | **p value** | **HR (95% CI)** | **p value** | **HR (95% CI)** |
| Highest SUVmax |  | 0.0021 |  | 0.0014 |  | 0.0054 |  | 0.0003 |  |
| < 7.0 | 51 |  | 1.00 |  | 1.00 |  | 1.00 |  | 1.00 |
| ≥7.0 | 41 |  | 3.59 (2.63-4.78) |  | 4.76 (3.21-9.89) |  | 3.65 (2.36-6.12) |  | 5.82 (3.52-16.97) |
| Total MTV (mL) |  | 0.0003 |  | 0.14 |  | 0.0005 |  | 0.26 |  |
| < 6.7 | 47 |  | 1.00 |  | 1.00 |  | 1.00 |  | 1.00 |
| ≥6.7 | 45 |  | 4.62 (2.71-8.92) |  | 1.63 (1.44-2.03) |  | 4.14 (2.79-8.78) |  | 1.27 (0.91-1.98) |
| Total TLG (g) |  | 0.0036 |  | 0.16 |  | 0.0077 |  | 0.27 |  |
| < 22.5 | 50 |  | 1.00 |  | 1.00 |  | 1.00 |  | 1.00 |
| ≥22.5 | 42 |  | 2.25 (1.58-3.27) |  | 1.54 (1.29-1.98) |  | 3.54 (2.32-6.14) |  | 1.25 (0.89-1.96) |
| PET N classification |  | 0.014 |  | 0.42 |  | 0.0001 |  | 0.56 |  |
| cN0 | 50 |  | 1.00 |  | 1.00 |  | 1.00 |  | 1.00 |
| cN1, N2, N3 | 42 |  | 1.89 (1.32-2.87) |  | 1.12 (0.85-1.45) |  | 3.64 (2.35-6.19) |  | 1.22 (0.85-1.48) |
| Clinical T classification |  | 0.0021 |  | 0.065 |  | 0.0017 |  | 0.19 |  |
| cT1, T2 | 83 |  | 1.00 |  | 1.00 |  | 1.00 |  | 1.00 |
| cT3, T4 | 9 |  | 2.26 (1.59-3.31) |  | 2.87 (1.91-3.98) |  | 3.72 (2.42-6.39) |  | 1.15 (0.89-1.59) |
| Pathological N classification |  | 0.016 |  | 0.80 |  | 0.0072 |  | 0.61 |  |
| pN0 | 44 |  | 1.00 |  | 1.00 |  | 1.00 |  | 1.00 |
| pN1, N2, N3 | 48 |  | 1.86 (1.29-2.79) |  | 0.91 (0.72-1.18) |  | 3.54 (2.22-5.86) |  | 1.16 (0.84-1.44) |
| Pathological Stage |  | <0.0001 |  | <0.0001 |  | <0.0001 |  | <0.0001 |  |
| Ⅰ,Ⅱ | 75 |  | 1.00 |  | 1.00 |  | 1.00 |  | 1.00 |
| Ⅲ | 17 |  | 5.36 (2.79-12.66) |  | 5.91 (3.52-18.58) |  | 5.75 (3.21-15.35) |  | 6.27 (3.84-26.43) |
| Histology |  | 0.99 |  |  |  | 0.45 |  |  |  |
| Invasive ductal carcinoma | 88 |  | 1.00 |  |  |  | 1.00 |  |  |
| Others | 4 |  | 0.61 (0.52-0.85) |  |  |  | 0.86 (0.58-1.31) |  |  |
| Tumor grade |  | 0.53 |  |  |  | 0.66 |  |  |  |
| 1, 2 | 52 |  | 1.00 |  |  |  | 1.00 |  |  |
| 3 | 40 |  | 0.84 (0.59-1.34) |  |  |  | 0.82 (0.62-1.31) |  |  |
| Ki-67 expression level |  | 0.23 |  |  |  | 0.29 |  |  |  |
| <20% | 20 |  | 1.00 |  |  |  | 1.00 |  |  |
| ≥20% | 72 |  | 1.19 (0.79-1.47) |  |  |  | 0.89 (0.63-1.49) |  |  |
| NAC |  | 0.0047 |  | 0.0037 |  | 0.41 |  |  |  |
| No | 30 |  | 1.00 |  | 1.00 |  | 1.00 |  |  |
| Yes | 62 |  | 2.21 (1.55-3.21) |  | 4.53 (2.91-7.16) |  | 0.86 (0.60-1.40) |  |  |

Abbreviations: SUVmax: maximum standardized uptake value, MTV: metabolic tumor volume, TLG: total lesion glycolysis,

PET: positron emission tomography, NAC: neoadjuvant chemotherapy, HR: hazard ratio, CI: confidence interval.
